# Support for the slip hypothesis from whisker-related tactile perception of rats in a noisy environment

**DOI:** 10.3389/fnint.2015.00053

**Published:** 2015-10-15

**Authors:** Christian Waiblinger, Dominik Brugger, Clarissa J. Whitmire, Garrett B. Stanley, Cornelius Schwarz

**Affiliations:** ^1^Systems Neurophysiology, Werner Reichardt Centre for Integrative Neuroscience, University of TübingenTübingen, Germany; ^2^Department of Cognitive Neurology, Hertie Institute for Clinical Brain Research, University of TübingenTübingen, Germany; ^3^Wallace H Coulter Department of Biomedical Engineering, Georgia Institute of Technology and Emory UniversityAtlanta, GA, USA

**Keywords:** behavioral modification, head-restraint rat, barrel cortex

## Abstract

Rodents use active whisker movements to explore their environment. The “slip hypothesis” of whisker-related tactile perception entails that short-lived kinematic events (abrupt whisker movements, called “slips”, due to bioelastic whisker properties that occur during active touch of textures) carry the decisive texture information. Supporting this hypothesis, previous studies have shown that slip amplitude and frequency occur in a texture-dependent way. Further, experiments employing passive pulsatile whisker deflections revealed that perceptual performance based on pulse kinematics (i.e., signatures that resemble slips) is far superior to the one based on time-integrated variables like frequency and intensity. So far, pulsatile stimuli were employed in a noise free environment. However, the realistic scenario involves background noise (e.g., evoked by rubbing across the texture). Therefore, if slips are used for tactile perception, the tactile neuronal system would need to differentiate slip-evoked spikes from those evoked by noise. To test the animals under these more realistic conditions, we presented passive whisker-deflections to head-fixed trained rats, consisting of “slip-like” events (waveforms mimicking slips occurring with touch of real textures) embedded into background noise. Varying the (i) shapes (ramp or pulse); (ii) kinematics (amplitude, velocity, etc.); and (iii) the probabilities of occurrence of slip-like events, we observed that rats could readily detect slip-like events of different shapes against noisy background. Psychophysical curves revealed that the difference of slip event and noise amplitude determined perception, while increased probability of occurrence (frequency) had barely any effect. These results strongly support the notion that encoding of kinematics dominantly determines whisker-related tactile perception while the computation of frequency or intensity plays a minor role.

## Introduction

Rodents rub their whiskers across objects to tactilely explore them. Long standing results in tribology (Bhushan, 2013), the field of mechanical interaction in relative object movement, predicts that relative movements of the elastic vibrissa across a texture, will complexly transform the function of space describing texture surface into hair vibration, a function of time, called the “vibrotactile signal”. In line with these predictions, high speed video recordings of moving whiskers revealed stick-and-slip movements (shortly called “slips” here)—kinematic signatures contained in the vibrotactile signal, defined by short-lived, fast deflections of the whisker. On sandpaper surfaces slip events last on average below 10 ms (with a heavy tail up to 40 ms), have a ramp-like appearance in position traces and are best visible as mono- or biphasic “humps” in velocity/acceleration traces (Ritt et al., 2008; Wolfe et al., 2008). Slips are based on bioelastic properties of the hair, i.e., its form and elasticity (Arabzadeh et al., 2005; Ritt et al., 2008; Hires et al., 2013), but have been reported to reflect properties of the probed texture as well (Wolfe et al., 2008; Jadhav et al., 2009). Different kinematic signatures are represented by highly selective spike responses on the ascending whisker-related tactile system (Jones et al., 2004; Arabzadeh et al., 2005; Petersen et al., 2008; Jadhav et al., 2009; Chagas et al., 2013). The slip hypothesis of perception states that information contained in slip frequency or kinematic profiles is (at least partly) used to construct the tactile percept of a touched texture (Jadhav and Feldman, 2010; Waiblinger et al., 2015).

The slip hypothesis provides constraints on the ways of how the vibrotactile signal should be analyzed to properly extract texture information. With rare and short-lived events as information carriers, measures to reject noise and strategies to detect the events within the noise are needed. In our mind, a good analogy is provided by “spike sorting”, a common procedure in neurobiology aimed to extract and classify extracellularly recorded action potentials buried in noise. Spike sorting commonly applies thresholding followed by some sort of shape recognition. These analyses operate near instantaneously, and thus, are well adapted to the scarcity and short duration of the target events. They provide guidance to create predictions for vibrotactile processing under the slip hypothesis. Most importantly, strategies aimed at classifying short-lived events must refrain from overly integrating the signal over large windows of time, because short events may get averaged out or may be masked by noise. This is in stark contrast to the kind of integration across large time windows required to arrive at signal “intensity”, or “best frequency”, two mainstays in current thinking on tactile perception with whiskers (Hipp et al., 2006; Ewert et al., 2008; Adibi et al., 2012) and finger tips (LaMotte and Mountcastle, 1975). Recently, approximating slips by passive pulsatile whisker deflections and combining the measurement of psychophysical performance and cortical neuronal spike activity has been employed to test the slip hypothesis. These studies yielded surprising support for the prediction that instantaneous signal processing should predominate temporal integration. Varying integration time of cortical spiking yielded best fits of neurometric and psychometric data when using integration windows in the range of tens of milliseconds, albeit, some temporal integration giving rise to inferior discrimination performance has been consistently found as well (Gerdjikov et al., 2010; Stüttgen and Schwarz, 2010; Waiblinger et al., 2015).

These previous findings have been obtained with pulsatile stimuli, which engage the tactile system exclusively during the stimulus pulses in a noise free environment. However, background activity (i.e., changed context) is likely to modify neuronal responses to specific kinematic signatures due to non-linear coding (Hentschke et al., 2006; Chagas et al., 2013) and/or adaptation (Maravall et al., 2007; Wang et al., 2010; Musall et al., 2014; Ollerenshaw et al., 2014). Therefore, it is important to find out, whether more natural pulse events, which, due to ongoing texture contact, are embedded in ongoing neuronal activity, could possibly be extracted by the same mechanism (we refer to them here as “slip-like” events to indicate the difference to real slips that occur with active object contact). To answer this question, we applied slip-like events within noisy background vibration of the whisker in operantly trained head-fixed rats. The noise amplitude used was deliberately chosen to be perceivable when presented by itself. We found that the rats were readily able to extract short slip-like events from background noise. Most importantly, rats predominantly used the kinematic signature of the slip-like events to detect them and largely failed to use the possible advantage to integrate the vibrotactile signal in case more than one slip-like event was presented.

## Materials and Methods

### Animals, Surgery, and General Procedures for Behavioral Testing

All experimental and surgical procedures were carried out in accordance with standards of the Society of Neuroscience and the German Law for the Protection of Animals. Subjects were seven female Sprague-Dawley rats (Charles River, Germany), aged 12–16 weeks at time of implantation. The basic procedures of head-cap surgery, habituation for head-fixation, and behavioral training exactly followed the ones published in a technical review (Schwarz et al., 2010). In the following only procedures pertaining to the special paradigm established here are described in detail.

Oral antibiotics (Baytril; Bayer HealthCare, Leverkusen Germany, 2.5% in 100 ml drinking water) were provided for 3 days before surgery and 1 week post-operatively. The animals were anesthetized using ketamine and xylazine (100 mg and 15 mg per kg body weight, respectively) and a screw for head fixation was implanted. The wound was treated with antibiotic ointment and sutured. Analgesia and warmth were provided after surgery. Rats were allowed to recover for at least 10 days before habituation training. Subjects were housed together with a maximum number of four in one group cage and kept under a 12/12 h inverted light/dark cycle. During testing, water intake was restricted to the apparatus where animals were given the opportunity to earn water to satiety. Testing was paused and water was available *ad lib* during 2 days a week. Body weight was monitored daily, and was typically observed to increase during training. No animal in this study needed supplementary water delivery outside training sessions to keep its weight. The first step of behavioral training was systematic habituation to head-fixation lasting for about two weeks. During behavioral testing a constant white background noise (70 dB) was produced by an arbitrary waveform generator (W&R Systems, Vienna, Austria) to mask any sound emission of the galvo-motor-based whisker actuator (see below).

### Whisker Stimulation

**Figure 1:**
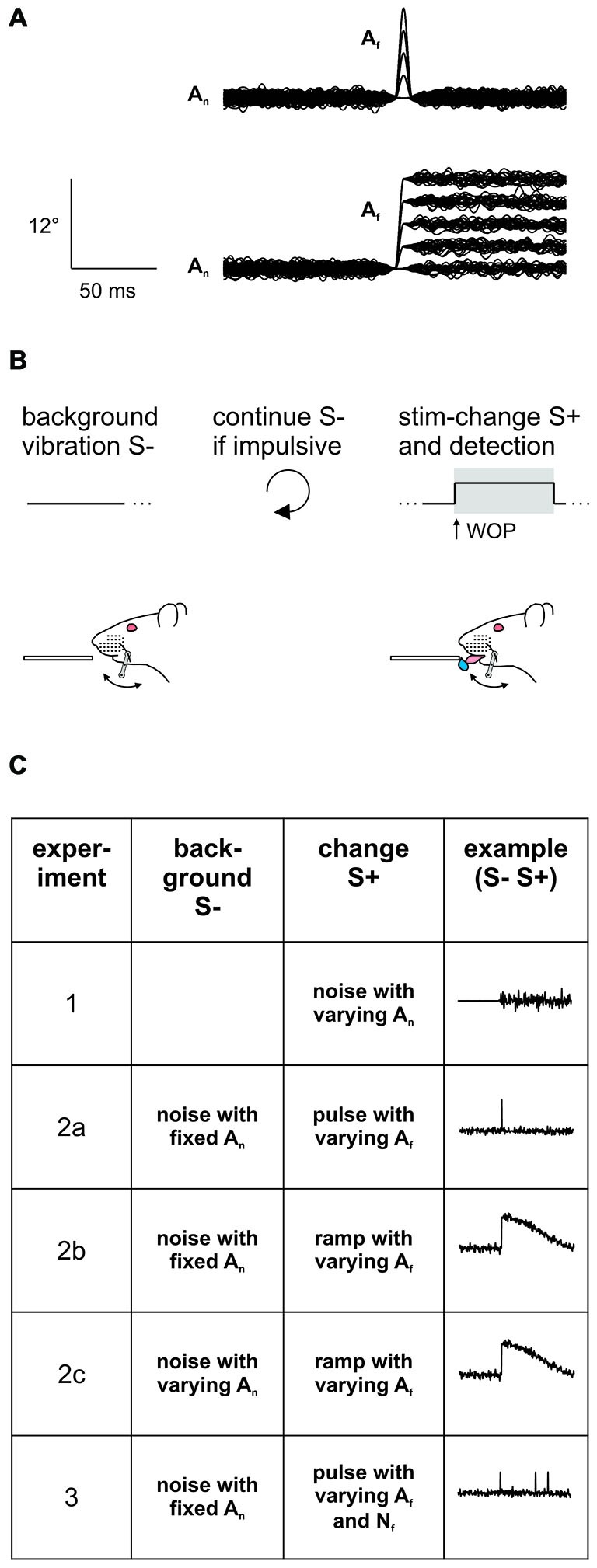
**Experimental strategy. (A)** Stimuli consisted in pulse- or ramp-shaped slip-like events of different amplitudes embedded into broadband noise (*n* = 100 trials overlaid in each panel). The noise was silenced with an inverse Gaussian at the time of feature location, thereby smoothing the fast transitions. Note the ramp was followed by a slow and imperceptible return to pre-ramp levels (better seen in Figure 3A; Stüttgen et al., 2006). **(B)** Psychophysical task. Head fixed rats were trained on a detection of change task (DOC). In this task the whisker is continuously vibrated using a background noise stimulus. A stimulus trial consists of a 1 s period in which slip-like events are embedded in the continuously ongoing noise stimulus (S+) (as in **A**). This change has to be detected and indicated by the animal by emitting a lick to gain a water reward (the window of opportunity (WOP), is the interval in which an indicator response elicits a reward. Here it is congruent with the stimulus period of 1 s). No change (no embedded events) served as catch trial. Impulsive licks during the inter-trial-interval triggered extra time of background vibration. **(C)** Overview of the stimulus sets applied in the different experiments of this study. In Experiment 1, perceptibility of broadband noise was assessed by presenting 1 s sections of different noise-levels (A_n_). In all the following experiments the noise served as background (S−) and was presented continuously throughout the entire session. In experiment 2 slip-like features (S+) of different amplitudes (A_f_) and shapes (pulses in experiment 2a or ramps in experiment 2b) were embedded exceeding the kinematics of the noise band. The noise amplitude was additionally varied in experiment 2c. In experiment 3 slip-like features of different amplitudes (A_f_) and numbers (N_f_) were embedded. See for details Table 1.

For whisker stimulation a galvo-motor (galvanometer optical scanner model 6210H, Cambridge Technology) as described in Chagas et al. (2013) was used. The stimulator contacted the whisker 5 mm (±1 mm tolerance) away from the skin, and thus, directly engaged the proximal whisker shaft, largely overwriting bioelastic whisker properties. The mean whisker position during noise stimulation was its resting point, with an angle between whisker and skin of about 90°. Stimulation was always delivered in rostro-caudal direction. Voltage commands for the actuator were programmed in Matlab and Simulink (The MathWorks, Natick, MA, USA). The whisker was deflected by Gaussian white noise (sampling rate 20 kHz) that was low-pass-filtered using a Butterworth filter (5th order) with cutoff frequency of 100 Hz (Chagas et al., 2013). The amplitude and velocity of these noise stimuli (A_n_, V_n_) was varied across different experiments and is indicated as 2SD of the respective distribution throughout this report (Table 1). The noise stimulus was presented continuously throughout the session. A stimulus trial could be of the type multi-event, single-event, or catch. It always consisted of a 1 s noise section (seamlessly continuing the background stimulus) and had no (catch), one, (single-event), or several (multiple event) slip-like features embedded into it. The feature amplitudes (A_f_ = [1.5, 3, 4.5, 6, 9, 12, 18, 24]° or maximal velocities respectively: V_f_ = [500, 1000, 1500, 2000, 3000, 4000, 6000, 8000]°/s^−1^) were well within the range reported for real slips (Ritt et al., 2008; Wolfe et al., 2008) and could vary from trial to trial. Whenever slip-like features were embedded, the first feature occurred at the start of the 1 s epoch. In multiple event stimuli, the following events were distributed at random within the 1 s epoch. The features consisted either of pulses (single-period sine wave; starting from the minimum, thus yielding a bell-shaped pulse with smooth on- and offsets; 100 Hz; duration 10 ms) or ramps (half-period sine wave; starting from the minimum, duration 5 ms with a slow decay of half period sinusoidal waveform; starting from the maximum, duration 995 ms). Ramp- and pulse-like whisker deflection was used in caudal and rostral direction in different sessions. We did not see any obvious difference in psychometric performance with the two directions. To assure a smooth embedding of these slip-like events, the noise was silenced (multiplied) with an inverted Gaussian (*SD* = 10 ms; minimum at the peak is 0, approaching 1 at ± infinity) centered at the time of the pulse peak or the time of the ramp’s maximum velocity. As a result, the fast transitions (pulse: up and down; ramp: up) where smooth and largely noise free (Figure 1A).

**Table 1:** **Overview of all experiments including stimulus parameters and number of trials for each animal**.

Experiment	Stim-type	A_n_ 2SD (°)	A_f_ (°)	N_f_	*N*-Trials						
					Rat 1	2	3	4	5	6	7
1	S−	0	−	−	156	228					
	S+	0.25	−	−	156	230					
	noise
		0.5	−	−	154	230					
		1	−	−	155	229					
		2	−	−	156	230					
2a	S−	1	−	−	226		132	165	174		
	S+	1	3	1	225		132	165	174		
	pulse
		1	6	1	225		130	165	175		
		1	9	1	225		131	167	175		
		1	12	1	226		134	167	175		
2b	S−	1	−	−	200	267				175	151
	S+	1	3	1	199	265				175	152
	ramp
		1	6	1	196	268				175	148
		1	9	1	199	266				174	150
		1	12	1	198	265				172	151
2c	S−	0.5	−	−	203						
		1	−	−	200						
		2	−	−	201						
	S+	0.5	1.5, 3, 4.5, 6	1	201*						
	ramp
		1	3, 6, 9, 12	1	196*						
		2	6, 12, 18, 24	1	200*						
3	S−	1	−	−		157	161	106	148		
	S+	1	3	1, 3, 6		155*	160*	105*	147*		
	pulse
		1	6	1, 3, 6		157*	159*	106*	145*		
		1	9	1, 3, 6		156*	161*	106*	149*		
		1	12	1, 3, 6		158*	162*	107*	145*		

*These numbers are minimum number of trials obtained for the S+ stimuli in the respective row. A_n_ = Noise Amplitude (2*SD). A_f_ = Feature Amplitude (base to peak). N_f_ = Number of features in train. Corresponding amplitudes and velocities of noise and features: A_n_ [0.5, 1, 2]° − V_n_ [175, 350, 700]°/s^−1^ (2*SD). A_f_ = [1.5, 3, 4.5, 6, 9, 12, 18, 24]° − V_f_ = [500, 1000, 1500, 2000, 3000, 4000, 6000, 8000]°/s^−1^ (base to peak).

### Experimental Paradigm

All seven rats were trained on a detection of change (DOC) psychophysical task (Waiblinger et al., 2015). In this task, the whisker is continuously vibrated, but vibration parameters change once in a while, an event that is to be detected (S+) and indicated by the animal by licking at a spout to gain a water reward (Figure 1B). Before data collection began, all subjects learned the DOC task employing the following protocol: In a first step, continuously applied broadband noise (S−, A_n_ = 1°) was interspersed every 4–10 s (random pick of inter-trial intervals from a flat distribution) by a rapid succession of strong slip-like features (S+, A_f_ = 12°, N_f_ = 6–20 pulses, each 10 ms duration, all occurring within a 1 s noise-section) automatically followed by the delivery of a water-drop to condition the consummatory response (licking) upon the slip-like stimuli. Once the animals regularly licked off the water, the task was switched from classical to operant conditioning, i.e., the reward delivery was made contingent on an operant lick during the occurrence of slip-like events plus extra 500 ms to allow for any temporal integration. Now, the rats were able to retrieve a water reward by licking once they detected one or multiple slip-like events. Licking during a “no-lick-interval” that spanned the last 2 s before the scheduled S+ presentation was mildly punished by resetting time and starting a new inter-trial interval with randomly picked duration. Catch trials (S−) always consisted in a seamless continuation of the stimulus presented during the inter-trial period. Responses in the catch period of 1 s were counted either as a false alarm (lick) or correct rejection (no lick), and did not have consequences (i.e., no reward/punishment).

For data collection psychophysical testing employed the method of constant stimuli which implies the presentation of stimuli in pseudo-random sequence. Pseudo-random order as applied here presented blocks in which all stimuli occurred once in randomly shuffled order (this strategy avoids sessions in which certain types of stimuli are presented toward the end or the beginning of the session by chance). The window of opportunity (WOP; in which an indicator response, a lick, would yield reward) was now restricted to 1 s to keep high false alarm rates (during catch trials) low. Three psychophysical experiments were conducted (overview in Figure 1C, details in Table 1).

The first experiment consisted of a presentation of 1 s broadband noise (S+) at four amplitudes (Table 1) at pseudorandom order against a background of whisker rest. The on- and offset of the noise stimulus was smoothed with a sinusoidal filter (50 ms duration) to avoid abrupt transitions. Continued whisker rest served as catch trial (S−). This control experiment was conducted to test the animals’ detection threshold for broadband noise.

In all other experiments, background vibration had non-zero amplitude and was applied constantly throughout the entire recording session whereas the embedded slip-like features (S+) occurred only in a trial based fashion (Table 1). Catch trials contained a continuation of the background noise (S−) without any embedded events, but the noise silencing used to embed the stimuli was kept to control for its possible function as a cue. In all experiments these catch trials were responded to with false alarm rates typical for this type of experiment (Waiblinger et al., 2015). Therefore, we conclude that the results of this study were not confounded by the noise silencing episodes.

In the second experiment slip-like events consisted either of single pulses (lasting 10 ms, Experiment 2a) or ramps (lasting 5 ms, Experiment 2b) at four different amplitudes, i.e., a total of five different stimuli including catch were presented in Experiments 2a and 2b respectively (Table 1). The slowly decaying part of the ramp in Experiment 2b (after the sharp upswing; duration 995 ms) did not offer an extra cue to the animal (Stüttgen et al., 2006) and was used to reset the stimulator to its zero position. Experiment 2c was like Experiment 2b, only that here three different blocks of varying background noise amplitudes were used. Across these three blocks, the feature amplitude was adjusted to keep the signal-to-noise ratio (A_f_/A_n_) constant. Across the sessions constituting Experiment 2c, a total of 15 different stimuli (including catch trials) were presented (Table 1).

The third experiment presented slip-like events containing three different pulse numbers, four pulse amplitudes and one catch trial resulting in 13 possible stimuli (Table 1). Pulses were presented within a maximal window of 1 s which also represented the WOP, in which an indicator response would yield a reward. The time window was always initiated by the first slip-like event (first pulse) and the following events were distributed randomly within the 1 s period with the only constraint that the inter-pulse-interval was always larger than 50 ms.

### Data Analysis and Statistics

Psychophysical data were assessed as response-probabilities for each animal, averaged across sessions. Error bars of psychometric data signify 95% confidence intervals calculated from a binomial model setting the animal’s response probability to the probability of a Bernoulli trial. The psychometric curves in this study are logistic fits estimated from a maximum likelihood estimator (Wichmann and Hill, 2001a,b). Statistical differences between psychophysical curves were assessed using 95% confidence limits of the thresholds (probability of detection = 0.5). Reaction times or lick delays were calculated by subtracting the timestamp of the first lick within the 1 s WOP from the onset of the respective slip-like event.

A Monte-Carlo resampling technique was used to model the detectability. Each resampling step consisted in constructing one of the stimuli as used in Experiment 3 (description above; this was repeated 1000 times for each stimulus). Each of these stimuli was convolved with a flat kernel of varying duration followed by thresholding, which gave us 1000 binary decisions (Go/NoGo) of the model from which we constructed model detection performance. The two free parameters of the model, thus, were integration time window and threshold which were varied systematically across a wide range. Best fits were identified by searching the minimal Euclidean distance of model performance to the measured performance of rats. In a second approach, we fitted a logistic model using the stimulus after filtering with the kernels of varying duration (as above) as the independent and the animal’s binary responses in *n* = 5119 trials (recorded in Experiment 3) as the dependent variable. Best fits were identified by assessing the mean deviance from the measured data, with deviance defined as the sum of the squared residuals (cf. Figure 5).

## Results

The present psychophysical data were sampled from seven rats each subjected to a DOC paradigm (Waiblinger et al., 2015; Figure 1B). Rat 1 and 2 were first trained on the detection of broadband noise (against no movement; Experiment 1) and then were subjected to a task in which they were required to detect multiple (N_f_ = 6–20) slip-like features as described in the methods section. All other animals (rats 3–7) were immediately trained on the latter task. In rats 3–5 the task was then refined for systematic psychometric assessment of feature amplitude A_f_ (velocity V_f_) and number (N_f_) as described in Experiment 3. In all rats the number of slip-like events was finally reduced to one (single ramp-like events for rats 1, 2, 6, 7; and single pulse-like events for rats 1, and 3–5; Experiment 2). All variations of the DOC task presented here were readily learned by the rats that were trained on them. For purposes of logic of presentation we will describe the single-event experiments (Experiment 2) before the multiple-event experiment (Experiment 3, in rats 2–4), although it is important to note they were actually performed in reverse temporal order.

**Figure 2:**
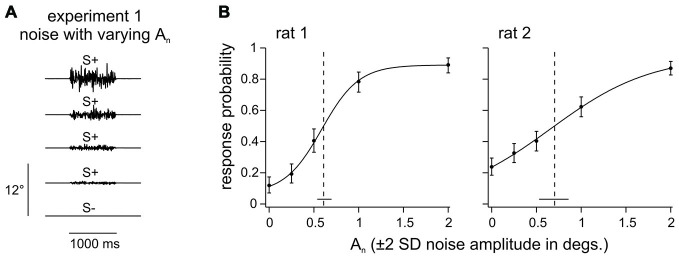
**Noise detection. (A)** 1 s of broadband noise with different amplitudes (A_n_) was presented pseudo randomly in a trial based fashion in Experiment 1. The task of the animal was to detect any whisker deflection. **(B)** Response probabilities of 2 rats are depicted as a function of noise amplitude (A_n_). Data points represent means (*n* = 154–230 trials per stimulus, 5–6 sessions) and smooth lines are logistic fits estimated from a maximum likelihood estimator. Vertical error bars represent 95% confidence intervals. Horizontal bars at the bottom represent 95% confidence intervals of the thresholds (dashed line).

Experiment 1 (rat 1–2) aimed at identifying a background noise level that is perceivable but does not saturate the sensory system. The two animals learned to detect the presence of a noisy whisker deflection of 1 s duration which varied in amplitude A_n_ (Figure 2A; A_n_ is given as ± 2*SD, thus, e.g., A_n_ = 1° indicates that, in 96% of the time bins, the stimulus position is within [−1°, 1°]). The psychometric curves fitted to these data indicated confidence intervals of the amplitude threshold of 0.54–0.70 degrees (rat 1) and 0.53–0.87 degrees (rat 2). Thus, a noise level of 1° would be readily perceptible and its location on the supra-threshold, sloped portion of the psychometric curve assures that it engages the tactile system without driving it into saturation (Figure 2B). The background noise amplitude was therefore set to A_n_ = 1° for all experiments in which this parameter was held fixed.

**Figure 3:**
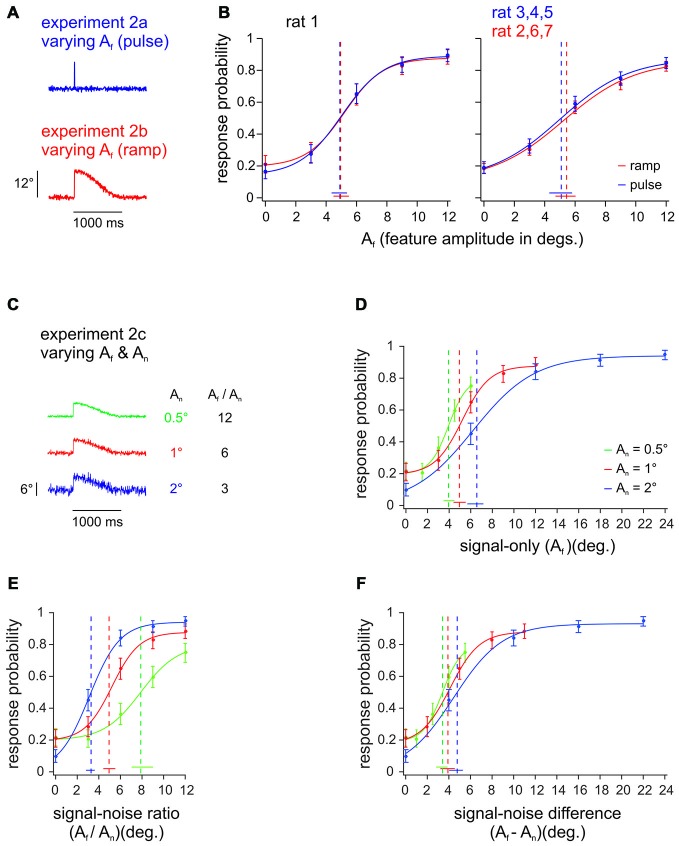
**Pulse vs. ramp detection. (A)** Slip-like features of different amplitudes (A_f_) and shapes, embedded into broadband noise. The events consisted either of pulses (single-period sine wave; 100 Hz; duration 10 ms, Experiment 2a) or ramps (half-period sine wave; duration 5 ms with 995 ms decay, Experiment 2b). **(B)** Left: Psychometric curves from one animal detecting pulse and ramp stimuli in alternating sessions (*n* = 196–226 trials per stimulus, 8–9 sessions). Right: Psychometric curves from 3 animals with pulse stimuli vs. 3 animals with ramp stimuli. Response probabilities are averaged across subjects and sessions (*n* = 427–593 trials per stimulus, 17–22 sessions). **(C)** In Experiment 2c the background noise level (A_n_) was varied between sessions (feature amplitude was adapted to keep signal to noise ratios constant). Here shown are 3 ramps with A_f_ = 6° and A_n_ = [0.5, 1, 2]° (out of 15 stimuli presented) exemplifying signal-to-noise ratios of 3, 6, and 12. **(D)** Psychometric performance of rat 1 to extracting slip-like features of different amplitudes. The curves represent psychometric functions obtained with features embedded in three levels of background noise (*n* = 196–203 trials per stimulus, 8–9 sessions). **(E)** Same data plotted as a function of the signal-to-noise ratio (A_f_ / A_n_). **(F)** Same data plotted as a function of signal-to-noise difference (A_f_−A_n_). Curve fit and error bar conventions as in Figure 2. Color in **(B)** refers to different feature waveforms (exemplified in **A**). Color in **(D–F)** refers to different noise amplitudes (exemplified in **C**).

Experiment 2ab (all rats) was designed to test whether it is feasible to use pulses to mimic natural slip events, which in fact rather take the form of ramps (Ritt et al., 2008; Wolfe et al., 2008). Toward this aim, we compared detection performance of ramps and pulses, which were identical in their upswing, but diverged in the downswing: Ramps would stay up and only slowly (and imperceptibly cf. Stüttgen et al., 2006) would decay back to zero position, while the fast downswing of pulses was mirror-symmetric to the upswing (Figure 3A, see Figure 1A for shorter timescale). This comparison carries considerable interest, as a classical finding was that one type of primary afferents, the slowly adapting variant (SA), responds quite differently to ramps and pulses. SA responses to ramps, consists in a tonic discharge long after the peak is reached (Gibson and Welker, 1983). SA responses to pulses or single periods of sinusoids, on the other hand, are single spikes or phasic bursts similar to the other known variant of primary afferents, the rapidly adapting cell (RA; Deschênes et al., 2003; Chagas et al., 2013). Despite this difference, rats’ detection of ramp-like deflections has been aligned best to the evoked SA spike rate, only if the tonic part of the SA responses was completely ignored, which led to the conjecture that tonic SA spikes are perceptually irrelevant (Stüttgen et al., 2006). This notion was confirmed by the finding that cortical responses were strongly phasic even for stimuli that evoked responses exclusively in SA primary afferents (Stüttgen and Schwarz, 2008). The decisive difference between SA and RA primary afferents may not be the presence of tonic firing with ramps, but rather their different responsiveness to different kinematic ranges of the vibrotactile signal (Stüttgen et al., 2006; Chagas et al., 2013). Equal detection of ramps and pulses would constitute another independent piece of evidence favoring the perceptual irrelevance of tonic SA spiking. One animal (rat 1) received both, pulses and ramps of identical amplitudes and maximal velocity, in alternating sessions (Figure 3B, left). The other rats were trained either on ramps or on pulses (Figure 3B, right; *n* = 3 for each group; rats 3–5 received pulses; rats 2, 6, 7 received ramps). As conjectured, the psychometric curves obtained with pulses and ramps were identical (given the precision of our measurement as indicated by the confidence intervals). In view of the extra spikes to ramps known to be generated by SA primary afferents, this result supports the notion that SA tonic spikes are irrelevant for perception (Stüttgen et al., 2006; Stüttgen and Schwarz, 2008). For the purposes of the present study (see Experiment 3), an important conclusion from this experiment is that the ramp-like slip events typically found in more natural conditions of whisker-object relative movement can be readily mimicked by pulses.

Before engaging in experiments with stimuli containing repetitive pulses, we wanted to test, how detection of slip-like events relates to the relative noise amplitude. Experiment 2c, thus, used varying noise amplitudes (0.5, 1, and 2°), and respectively scaled slip-like feature amplitudes and velocities (rat 1, Figure 3C shows the three out of 15 stimuli with A_f_ = 6°, exemplifying signal to noise ratios of 3, 6, and 12). In a first approach we plotted the psychometric curves across A_f_ (Figure 3D). Non-overlapping 95% confidence intervals of detection thresholds indicate that the psychometric performances on stimuli with different noise levels are significantly different, matching the common intuition that signals embedded in higher noise are more difficult to detect. This finding suggests that the animal would show different detection performance also for different signal to noise ratios (A_f_/A_n_)—a conclusion that was confirmed by plotting the same behavioral data across A_f_/A_n_ (Figure 3E). In fact, it appeared as if the animal detected high amplitude stimuli better, partly overcoming the influence of the higher noise amplitudes increased by the same factor. The blue curve contained the highest absolute stimulus amplitudes and was significantly shifted left (non-overlapping threshold 95% confidence intervals) with respect to the other curves containing lower absolute stimulus amplitudes. This finding is remarkable as it does not support the notion that the animal used a simple stimulus integration scheme to arrive at its perceptual decision. If rats integrated the presented stimuli over long windows, their performance should match across identical signal-to-noise ratios independent of noise levels—an expectation that was clearly violated by our findings.

In contrast, the slip hypothesis would predict that rare and short lived kinematic events are the basis for detection and discrimination. We reasoned that detection of such events would be well served by a thresholding procedure that would reject noise (i.e., reduce the probability of false alarms) and allow the perceptual system to focus on the infrequent, large amplitude kinematic events. Such thresholding could be realized e.g., by neuronal adaptation (Maravall et al., 2007) which effectively would adapt the responses to noise amplitudes away and amplify responses to rare deviant stimuli (Wang et al., 2010; Musall et al., 2014). If this were the case, perception should be related to the difference of signal and noise amplitudes rather than to their ratio. Indeed, plotting the same behavioral responses across the difference between event and noise amplitudes (A_f_−A_n_) corroborated this hypothesis, as it revealed aligned psychometric curves with overlapping threshold 95% confidence intervals (Figure 3F).

**Figure 4:**
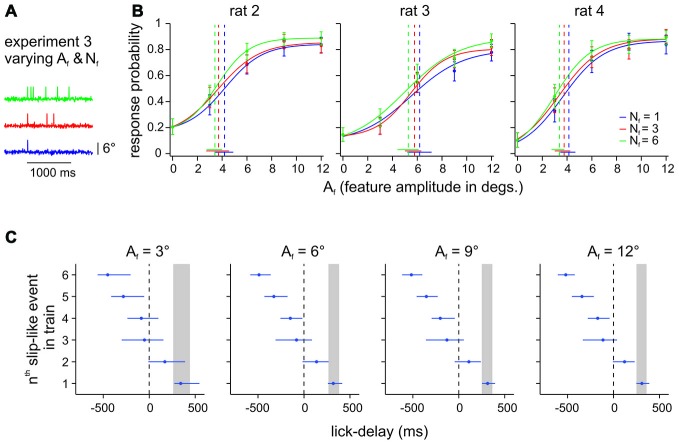
**Effect of number of slip-like events (N_f_) on perception. (A)** Example trials from Experiment 3 with different N_f_. Slip-like events were presented within a maximal window of 1 s which also represented the time window for response and potential reward. The time window was always initiated by the first slip-like event and the following events were distributed randomly with a minimal distance of 50 ms peak to peak. **(B)** Psychometric curves of 3 animals performing the DOC task with slip-like features varying in number (N_f_) and amplitudes (A_f_). Each data point represents the mean response probability as a function of feature amplitude A_f_ (*n* = 105–164 trials per stimulus, 10–13 sessions). Curve fit and error bar conventions as in Figure 2. **(C)** Median lick delays and interquartile ranges averaged across animals for all stimuli (sub-panels separate different slip-like feature amplitudes A_f_). Lick delays to single events from Experiment 2 are also shown for comparison (the inter-quartile range is indicated by the gray box).

The psychometric curves obtained in Experiment 2 clearly established that rats can readily detect single slip-like events embedded in noise (Hit rates were typically found above 0.8 and FA rates of ~0.2 (Figure 3). We next tested the perceptual capabilities of 3 animals (rat 2–4) to extract slip-like events of different amplitudes and different numbers (frequency) from the background noise (Figure 4A). The animals were allowed to immediately report the first slip-like event after it had occurred. (As noted before, all animals received initial training using multiple pulses before being subjected to Experiment 3 to provide them with the possibility to learn to use temporal integration, if they could. Single event testing as needed for Experiment 2 always came last in the training sequence). If the animals integrated across slip-like events, we would expect higher response rates for trials with a higher event number and prolonged reaction times. However, the presence of multiple slip-like events (red and green curves in Figure 4B) only slightly improved Hit rates above the ones observed with single slip-like events (blue curves) with a non-significant increase of perceptual thresholds (as indicated by overlapping 95% confidence intervals). Evaluation of lick delays (interval between a slip-like event and the rewarded lick) further revealed that a majority of successful licks were hardly affected by event number. Figure 4C plots the inter-quartile ranges of lick delays averaged across animals for the first, up to the 6th slip-like event. The expected lick delay (inter-quartile range) as estimated from the detection of single slip-like events (cf. Experiment 2a, above), is gray-shaded, and matches very well the typical range as has been measured in detection tasks using single pulses (Stüttgen and Schwarz, 2010). The plot reveals that the lick delays for detection of multi-slip-like stimuli falls within the range of those observed with single slip-like events. Only in rare cases the animals might have responded to the second slip-like event (the lick time distribution overlapped somewhat with the one seen with single slip-like event), while lick times relative to the third event and later are mainly negative, i.e., the animals regularly had responded before they occurred. We conclude that although the animals have been trained on multiple slip-like events, they do not integrate the vibrotactile signal to optimize perception.

## Discussion

The present report provides behavioral evidence that rats are readily able to detect slip-like events embedded into noise. We show that rats use near instantaneous kinematic aspects of the slip-like events rather than evaluating their number. Thus, temporal integration across the noisy signal using wide integration intervals plays a minor role for detection.

**Figure 5:**
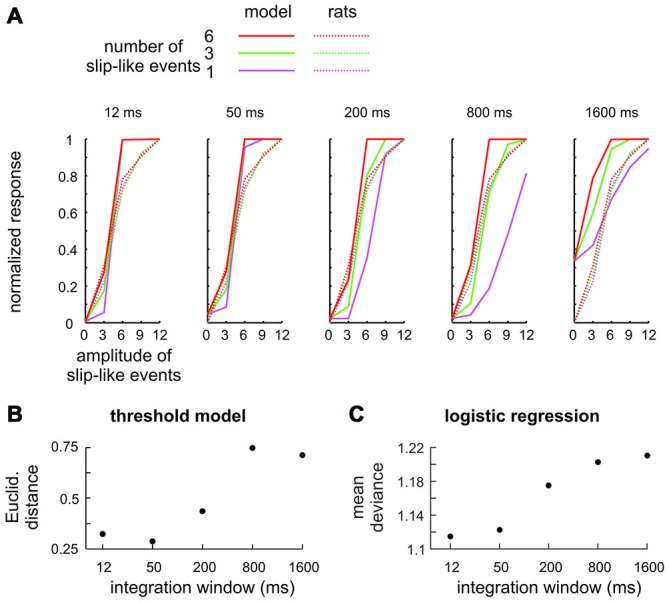
**Integration model to detect slip-like events. (A)** Signal integration with different temporal filters (boxcar filters) was subjected to a variable threshold to classify trials into Hits (slip-like events present and detected) and Misses (slip-like events present but not detected). The model performance (full lines) using the threshold yielding the minimal Euclidean distance to the rats’ performance is shown for the 5 integration windows together with the actual mean perceptual performance of the rats (broken lines). **(B)** Comparison of detection thresholds between model and actual performance of the rats. The best fit (minimal Euclidean Distance) was obtained with short integration windows. **(C)** Logistic regression between the integrated stimulus trace and the animals’ decision. The estimated mean deviance (mean of the squared residuals) is shown for models using different integration windows. As in **(B)** the best fit was obtained with short integration windows.

It could be argued that the stimuli presented here are not ideal to prompt detection using integration as slip-like events are presented at relatively low frequency. A simple modeling exercise shows that this objection is unfounded. We built a series of models which systematically varied the temporal integration time, modeled by convolving the stimuli used in the experiments with flat kernels (boxcar filters) of varying duration. A first approach aimed at a comparison of model performance with psychometric data, and therefore, explicitly calculated the “model’s decision”. To this end a second model parameter was used—a threshold applied to the filtered stimulus above which the model generated a “detect” response (classified as Hits or Misses). The model best fitting the animals performance (full lines), obtained with this strategy, is shown for each window duration and is compared to the mean perceptual performance of rats 2–4 (broken lines) in Figure 5A. All models showed some ability to detect the stimuli. However, the models using small integration windows, i.e., the ones tuned to near instantaneous signatures, fit the behavioral data best, as they reproduced the similarity of psychometric curves across the number of presented slip-like events (different colors). The similarity (averaged across number of slip like events) is expressed as Euclidean distance in Figure 5B. The second approach avoided the perhaps unrealistic assumption that responses are generated by applying a fixed threshold to the stimulus and was built instead on the assumption that psychometric curves are based on the logistic function, the S-shaped curve expressing a probabilistic neuronal contribution to the individual’s responses. This second series of models used only one free model parameter, the window duration, and logistically regressed the filtered stimulus and the animals’ decisions (Hit vs. Miss). The result of this approach was comparable to that of the first one—the best logistic fit (minimal mean deviance) was found with models using the smallest windows (Figure 5C).

In conclusion, the modeling results firstly abolish doubts that with a strategy of integration the rats might not have been able to effectively detect the stimuli, and secondly, lend further support to the hypothesis that the rats in fact used instantaneous coding to detect slip-like events in noise. The dominance of near instantaneous encoding of slip-like kinematics is in line with a wealth of data indicating that the whisker-related tactile code is fast. Primary afferents encode 10 ms long snippets of the vibrotactile signal at highest precision (Chagas et al., 2013). Available evidence suggests that such near instantaneous encoding is preserved up to the VPM thalamus and barrel cortex. Kernel based encoding models, whenever applied to tactile stations on the ascending pathway, have uniquely revealed short-lived features resembling the known slip waveforms (Jones et al., 2004; Maravall et al., 2007; Petersen et al., 2008; Chagas et al., 2013). Fittingly, perceptual measurements have shown that integration of pulsatile stimuli is neither dominant for detection nor for discrimination performance (Stüttgen and Schwarz, 2010; Georgieva et al., 2014; Waiblinger et al., 2015).

Available behavioral evidence in favor of temporal stimulus integration, to our knowledge, all contain ambiguities which allow the interpretation in terms of integration or instantaneous kinematic events. The sinusoidal stimulus in principle cannot differentiate between instantaneous coding and signal integration because frequency modulation always involves concomitant changes in the distribution of kinematic parameters (e.g., LaMotte and Mountcastle, 1975; Adibi et al., 2012, see detailed discussion in Waiblinger et al., 2015). A neurophysiological study using repetitive pulsatile whisker stimulation observed that neuronal responses to stimuli with varying (“noisy”) pulse amplitudes are larger than those to stimuli containing constant pulse amplitudes (Lak et al., 2010). However, in this study responses to individual pulses showed also a clear tuning toward higher pulse amplitudes (or velocities) which in principle is as well compatible with instantaneous evaluation of pulse amplitudes to be able to discriminate noisy from non-noisy stimuli. Despite the dominance of near instantaneous encoding found in the present study it is noteworthy that in other studies that have tried to disambiguate coding of integrated stimulus vs. near instantaneous parameters, always a certain degree of stimulus integration as basis of perceptional performance has been found (Gerdjikov et al., 2010; Stüttgen and Schwarz, 2010; Waiblinger et al., 2015). Future work is needed to identify the behavioral context that determines the usage of stimulus integration as a basis of perception.

In the present study we showed that near instantaneous encoding of kinematic signatures is used also in the presence of noise. This is important as neuronal adaptation to noise decisively changes stimulus responses (Fairhall et al., 2001; Maravall et al., 2007). The possibility that the signal to be detected is short-lived, however raises the question how exactly neuronal adaptation modulates the relationship of signal to noise. The classical version of signal-to-noise ratio (employed for instance in electrical engineering) relates the power of signal to that of the noise (power is proportional to the mean squared). If the task is to detect a short-lived signal embedded in noise, operating with means (i.e integrating the signal) may not be the most promising strategy. A more promising strategy would be to threshold the signal and focus on those parts of the signal exceeding the noise (a common strategy to detect rare events in noise, e.g., action potentials in extracellularly recorded neurophysiological signals). If the effect of neuronal adaptation is to realize a thresholding operation, then the relationship between amplitudes of signal to noise should be akin to subtraction rather than to division. Our finding that psychometric curves are different when plotting them across signal-to-noise ratios and become indistinguishable when plotted over the difference of signal and noise amplitudes supports this hypothesis. (Note our version of signal-to-noise ratio is different from the classical definition in engineering. It is an instantaneous one as we use the amplitude at peak as our measurement of signal amplitude). Our findings are paralleled by physiological research on neuronal adaptation, which showed that characteristics of adaptation may be suited for this task: spike rates to different ongoing noise amplitudes tend to be equalized, thus effectively “adapting away the noise” (although adapted spike trains keep some subtle scaling of absolute signal amplitude, cf. Figure 2 in Maravall et al., 2007), and shows a relative amplification of spike rates in response to rare deviant stimuli (Wang et al., 2010; Musall et al., 2014). Future work on the neuronal mechanisms of adaptation should manipulate noise and signals independently and in systematic ways to better describe neuronal adaptation effects on perception.

## Author Contributions

CW conceived experiments, conducted experiments, analyzed data, wrote the paper. DB conducted experiments. CJW conceived experiments, analyzed data, wrote the paper. GS conceived experiments, wrote the paper. CS conceived experiments, analyzed data, wrote the paper.

## Conflict of Interest Statement

The authors declare that the research was conducted in the absence of any commercial or financial relationships that could be construed as a potential conflict of interest.
